# Discovery of Possible Gene Relationships through the Application of Self-Organizing Maps to DNA Microarray Databases

**DOI:** 10.1371/journal.pone.0093233

**Published:** 2014-04-03

**Authors:** Rocio Chavez-Alvarez, Arturo Chavoya, Andres Mendez-Vazquez

**Affiliations:** 1 Department of Information Systems CUCEA, Universidad de Guadalajara, Zapopan, Jalisco, Mexico; 2 Department of Electrical Engineering and Computer Science Campus Guadalajara, Cinvestav, Zapopan, Jalisco, Mexico; Queen's University Belfast, United Kingdom

## Abstract

DNA microarrays and cell cycle synchronization experiments have made possible the study of the mechanisms of cell cycle regulation of *Saccharomyces cerevisiae* by simultaneously monitoring the expression levels of thousands of genes at specific time points. On the other hand, pattern recognition techniques can contribute to the analysis of such massive measurements, providing a model of gene expression level evolution through the cell cycle process. In this paper, we propose the use of one of such techniques –an unsupervised artificial neural network called a Self-Organizing Map (SOM)–which has been successfully applied to processes involving very noisy signals, classifying and organizing them, and assisting in the discovery of behavior patterns without requiring prior knowledge about the process under analysis. As a test bed for the use of SOMs in finding possible relationships among genes and their possible contribution in some biological processes, we selected 282 *S. cerevisiae* genes that have been shown through biological experiments to have an activity during the cell cycle. The expression level of these genes was analyzed in five of the most cited time series DNA microarray databases used in the study of the cell cycle of this organism. With the use of SOM, it was possible to find clusters of genes with similar behavior in the five databases along two cell cycles. This result suggested that some of these genes might be biologically related or might have a regulatory relationship, as was corroborated by comparing some of the clusters obtained with SOMs against a previously reported regulatory network that was generated using biological knowledge, such as protein-protein interactions, gene expression levels, metabolism dynamics, promoter binding, and modification, regulation and transport of proteins. The methodology described in this paper could be applied to the study of gene relationships of other biological processes in different organisms.

## Introduction

A number of methods have been applied over the years in an attempt to uncover the relationships among genes. One of these methods involves the modeling of gene relationships as Boolean networks, in which the state of a gene is represented as being “off” or “on” [1]. Even though these models are easy to interpret, it is usually difficult to determine the best way to convert gene expression levels into discrete values; moreover, there can be loss of information during the discretization process, which may affect the inference outcome. Static and dynamic Bayesian methods have also been applied by inferring the causal relationship between two network nodes based on conditional probability distributions [2], [3]. Differential equations [4], [5] and computational intelligence approaches, such as genetic algorithms [6], genetic programming [7], neural networks [8], and fuzzy logic [9] have also been applied. Reverse engineering of regulatory networks has also been explored [10], [11], [12]. However, the genetic regulatory network models obtained with different approaches tend to differ, without being able to reach a consensus on which of them is the most accurate; moreover, central assumptions of some models are not supported by experimental evidence [12], [13].

The pattern recognition technique known as Self-Organizing Map (SOM) is a type of unsupervised artificial neural network developed by Teuvo Kohonen [14]. The SOM algorithm performs mathematical cluster analysis useful for recognition and classification of features in complex, multidimensional data without using prior knowledge. It has been applied by Tamayo et al. [15] to extract genes involved in cell-cycle regulation from one database provided by Cho et al. [16], achieving similar results to those obtained by the latter. In the present work we propose the use of SOM for the study of gene interactions using DNA microarray data as input.

We applied the SOM technique to five of the most cited *Saccharomyces cerevisiae* cell cycle databases in order to predict the possible relationships among the genes involved in the cell cycle process. For over fifteen years, numerous researchers have directed their efforts at elucidating the way gene interactions in *S. cerevisiae* control its cell cycle in order to obtain knowledge about structural, biochemical, physiological and behavioral characteristics of this organism.

One of the biggest obstacles for performing mathematical inferences of the gene regulatory networks involved in the cell cycle regulation of *S. cerevisiae* is the small quantity of samples contained in the existing databases. D’haesseler et al. proposed the use of interpolation of time points between the existing samples to increase the quantity of measurements [17], but there is no certainty that the interpolated points represent the real behavior of genes between the known time points. Other proposals suggested the merging of several time series microarray databases to perform an analysis of cell cycle periodicity of gene expression [18], [19]; however, most databases have different sampling intervals of time, which makes it difficult to combine the expression levels contained in them. Furthermore, the conditions for the experiments or the strains of *S. cerevisiae* used in them are also different. It is assumed that the basic physiology of such strains will be the same, and therefore the results obtained with one strain will be applicable to others; however, Gaisne et al. remark that different *S. cerevisiae* strains present polymorphisms in restriction sites and chromosome size, and that the modification or inactivation of a regulatory factor probably affects the pathways in which it is involved [20], which could be one of the reasons why even when the same inference method is applied to the same set of genes in different databases, the regulatory networks obtained are very dissimilar. Instead of combining the gene expression levels from the different datasets to perform the analysis of the behavior of genes during the cell cycle process, we propose to find possible regulatory relationships based on the consistency of gene co-expression patterns in the five time series microarray databases, since genes that are co-expressed throughout a variety of conditions may be controlled by a common regulatory system [21].

Using the SOM algorithm, it was possible to detect genes whose products have possible biological and regulatory relationships among some of them, either directly or indirectly, through the consistency of the artificial neuron distances between genes in all five of the databases. These results were compared with a regulatory network provided by Alberghina et al. [22] –which has been determined mainly through biological methods– in order to validate the proposed methodology. Some examples of gene clusters consistently found in the five microarray databases are presented in the Results and Discussion Section. This section also discusses the usefulness of the color maps produced by SOM –which represent the intensity of gene expression during each cell cycle phase– in the determination of possible gene function and their importance in the cell cycle phase in which they show the highest expression level.

The analysis presented in this paper was useful in discovering possible relationships among genes using relatively few time points, based on their consistent behavior over time in the five databases of the *S. cerevisiae* cell cycle, without any prior knowledge about their characteristics, such as biological function or binding sites contained in them. We also present the analysis of gene pairs that showed similar behavior in four of the five databases, but that nevertheless behaved in a different way in the fifth database. The discovery of these outliers suggests that the different experimental conditions under which the dissenting database was obtained may affect the expression levels of these particular genes.

## Method

Five databases containing information on expression levels from *Saccharomyces cerevisiae* along two cell cycles were analyzed with theSOM algorithm. We chose these databases as a test bed for the proposed method, as all of them have been widely used over the years by different authors. These databases were obtained under different experimental conditions (see Table 1). Each experiment starts with cell populations that have been synchronized using different methods that arrest the cells in M/G1 phase. The *alpha* and *cdc15* databases were obtained by Spellman et al. using non-commercial spotted DNA/cDNA microarrays platforms identified as GPL59 and GPL62 in the NCBI GEO database. These two databases contain temporal expression levels for 6178 ORFs of *S. cerevisiae*. Data from microarrays used for the *alpha* database experiment were estimated to be missing <1% of all elements, whereas those used for the *cdc15* database were estimated to be missing ∼3% of all elements. In the *alpha* database experiment, yeasts were first arrested with the α-factor, then the α-factor was washed out, and cells were released into fresh medium. The *alpha* database contains 18 samples taken every 7 minutes, whereas the *cdc15* database contains 24 samples taken every 10 or 20 minutes [18]. The *cdc28* database contains 17 samples taken every 10 minutes and was obtained by Cho et al. [16], who used Affymetrix cDNA microarrays, containing probes for 6218 genes. This database does not appear in the NCBI GEO database. The three databases mentioned above can be obtained at http://genome-www.stanford.edu/cellcycle/data/rawdata, where they are provided as a tab-delimited data file. The accession number in the NCBI GEO database for the raw data of the databases from Spellman et al. are GSE22 for the *alpha* database and GSE23 for the *cdc15* database. The *alpha30* and *alpha38* databases were obtained by Pramila et al. and are biological replicates, in which cells were arrested in the G1 phase, with 25 samples each taken every 5 minutes [19] using cDNA microarrays prepared as described in [23] and containing 6229 ORFs. The accession number for both microarray datasets in the NCBI GEO database is GSE4987, whereas the microarray platform is identified in this database as GPL1914. For the experiments from which the *cdc15* and *cdc28* databases were obtained, cell growth was synchronized using temperature mutants, whereas in the rest of the databases cells were synchronized using the α-factor as mentioned above.

**Table 1:** Experiment conditions and yeast strains used in the five databases analyzed.

Database name	alpha	cdc15	cdc28	alpha30	alpha38
***S. cerevisiae*** ** strain**	DBY8724	DBY8728	K3445	BY2125	BY2125
**Characteristics**	MATa GAL2 *ura3 bar1*::URA3	W303α *cdc15-2^ts^* Temperature mutant	*cdc28-13* W101 Temperature mutant	W303: *MATa ade2-1 trp1-1 can1-100 leu2-3*, *115 his3-11 ura3 ho ssd1-d*	W303: *MATa ade2-1 trp1-1 can1-100 leu2-3, 115 his3-11 ura3 ho ssd1-d*
**Culture temperature**	25°C	23°C, 37°C, 23°C	25°C, 37°C, 25°C	30°C	30°C
**Cell cycle synchronization method**	α-factor	Temperature variations	Temperature variations	α-factor	α-factor
**Number of samples**	18	24	17	25	25
**Sampling time (min)**	7	10 or 20	10	5	5

The analysis was performed on 282 genes reported by Alberghina et al. [22], which have shown an activity during cell cycle phase changes in experiments conducted at the laboratory. These genes are part of a regulatory network that was determined using biological knowledge, such as protein-protein interactions, gene expression levels, metabolism dynamics, promoter binding, and modification, regulation and transport of proteins. A list of the genes from Alberghina et al. is provided as supporting information both in XLS and CSV format in Files S1 and S2, respectively.

It should be noted that the emphasis of the present paper is on the application of a pattern recognition method for comparing gene expression databases obtained under different experimental conditions, and not on the analysis of the particular *S. cerevisiae* genes chosen to validate the methodology. We propose the use of SOM and the measure of the distance among its output map units for the analysis of the degree of similarity among genes within each database. For the application of SOM, the MATLAB SOM Toolbox provided by Vesanto et al. was used [24], which has a variety of choices to perform the analysis and to display the results.

### Self-organizing Maps

Self-Organizing Maps are unsupervised neural networks that spatially organize high dimensional information into a one-, two- or three-dimensional output grid, in such a way that similar input data are mapped to areas that are closer to each other than the more dissimilar ones [14]. It can be said that the grid that the SOM technique outputs is both a similarity graph and a clustering diagram. SOM makes complex information abstractions and shows them in a very simple and visual way [25]. The main limitation of this algorithm is that there is no theoretical foundation for determining the parameters, such as the number of clusters to be used, the number of iterations to be performed, or the output neuron shape, that yield the best results. It might take a number of empirical tests until the appropriate parameters can be determined.

**Figure 1:**
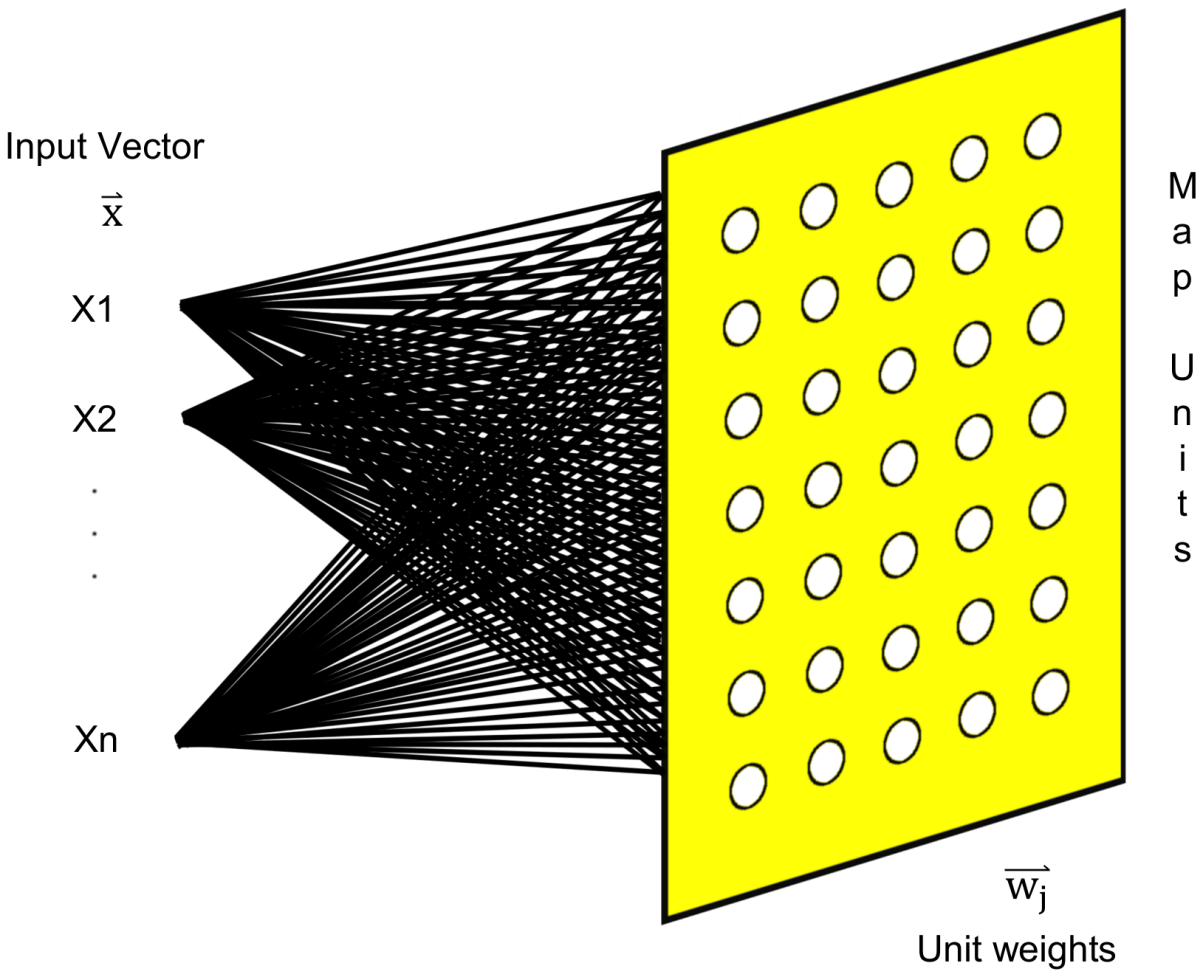
Input and output connections in the neural network of a Self-Organizing Map. An input vector (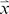) is defined by the expression levels of the gene to be analyzed. Each input vector is compared iteratively with the weights of the *j*-th output neuron (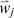), searching for the neuron with the highest similarity with the gene behavior along the time course, and organizing all the genes on the map according to their degree of similarity.

As in supervised neural networks, in SOMs each set of properties from one sample forms an input vector. Each input vector is connected to all the units on the output map, but the neural network used by the SOM has no intermediate layers (see Figure 1). The units on the output map have a weight, assigned initially either randomly or through the use of the eigenvectors corresponding to the two largest principal components of all input vectors [25]. In the present work, initial weights were assigned using the latter method.

By using the principal components method to initialize each unit’s weight, a two-dimensional space is created based on the two greatest eigenvalues and their eigenvectors, which are calculated from the input data. If the initial weights are not chosen properly, the SOM could generate a sub-optimal clustering partition [26]. The principal components initialization method can reduce the converging time of the SOM algorithm by several orders of magnitude [25], which can have a noticeable impact shortening execution times when analyzing large amounts of input data.

The SOM algorithm is performed in three main steps: the competitive process, the cooperative process, and the synaptic adaptation process.

### Competitive Process

This process is carried out through competitive learning. Only one output neuron can win: the vector that has the maximum similarity with the input vector. The winning vector is called the Best Matching Unit (BMU). An input vector contains the expression levels for all time samples for one gene. Maximum similarity can be calculated with different functions, such as the Euclidean distance, the inner product, or the Mahalanobis distance functions, among others. The Euclidian distance function was used in the present work, as Kohonen recommends this function for natural signal patterns corresponding to metric vector spaces [27]. The Euclidian distance function is defined by.
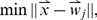
(1)where 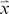 is the input vector and 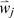 is the vector of unit weights for the *j*-th neuron on the output map. The input vector corresponding to the time series of expression levels of one gene is compared against all the neurons in the output map in order to find the neuron with which it has the maximum similarity.

The BMU weight is adjusted as described below to obtain even more similarity with the input vector. In this manner, the possibility of being the winning vector during the competitive process on the next iteration is increased in order to be the neuron that represents the compared input vector on the output map.

### Cooperative Process

The purpose of this process is to calculate which of the non-winning units are within the BMU’s neighborhood. The weights of these units are also adjusted, but in proportion to their proximity to the BMU, whereas the weight of the units outside the neighborhood is left intact. In order to find the neighbor units, it is necessary to set an initial neighborhood radius, which will be monotonically shrinking throughout the iterations. Some neighborhood functions that can be used to calculate the neighborhood radius are the Mexican hat, the rectangular, and the Gaussian functions. In the present work the Gaussian function was used, as it is the most commonly used neighborhood function in SOMs. The Gaussian function is given by.
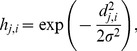
(2)where 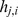 is the topological neighborhood value of unit *j* centered around the winning unit *i*, 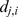 is the lateral distance between the winning unit *i* and the neighbor unit *j*, and σ is the decreasing neighborhood radius value.

### Synaptic Adaptation Process

In order to keep the neural network from overfitting, a forgetting term of what has already been learned is introduced. This term is based on the Hebbian hypothesis and is called the learning rate (*η*). This term also decreases throughout the iterations.

### Unit Weight Adjustment

Every unit on the output map adjusts its weight using the function.
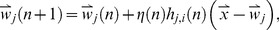
(3)where *n* is the iteration number, and the rest of the terms are as previously described. The greater the proximity of neuron *j* with the winning neuron *i*, the higher the value of the neighborhood function *h_j,i_(n)*, which results in a better adjustment on the weight of the neuron, as opposed to those that are farther away from the winning neuron. When the neuron is the BMU (*j = i*), the neighborhood function *h_j,i_(n)* value is equal to 1, so that the difference between the input vector and the weight of the BMU multiplied by the learning rate *η (n)* is added to its current weight. In this manner, the BMU becomes increasingly similar to the input vector that is being compared. The desired behavior is that neuron values on the output map are similar to the input data, and additionally that the input vectors are placed on the output map according to their similarity.

After calculating the unit weight for all the input vectors, the learning rate *η(n)* and the neighborhood radius (σ) are decreased and the iteration number is increased. The output map weights are gradually adjusted to resemble the input data and reflect its properties as closely as possible [28].

As for the parameters used in the SOM runs, the shape of the output map was defined as a sheet with a hexagonal lattice of 20 by 20 units, the neighborhood function was Gaussian with an initial radius of 3, and a decreasing learning rate type ‘inv’ was used as implemented in the MATLAB SOM Toolbox. The total number of iterations in the algorithm was defined as 10 multiplied by the number of neurons on the map divided by the number of genes to be analyzed.

### Data Preprocessing

Due to the lack of standardization in the labeling of gene names within the different microarrays (some of them use the standard name, some use the systematic name, and some use the gene alias), it was necessary to match the gene names in all the databases. We used the alias names contained in the databases from Spellman for the name standardization of the databases. Genes with missing data in more than two time points within each database were excluded from the present study (see Table 2). Missing data were calculated using an iterative algorithm called Expectation Maximization, which estimates the parameters pertaining to the data distribution, taking into account the mean and the variance. After this step, the missing data that best fitted that distribution was calculated. All the databases selected were already normalized, but due to the fact that some of the databases were in *log_2_* and others in *log_10_*, values were transformed into expression level fractions in order to make them comparable. This transformation preserves the original relationship between variables and produces no change in their probability density function. The five databases scaled to the range [0,1] are provided as supporting information (see Datasets S1, S2, S3, S4, and S5). A compressed file with the SOM Toolbox files used for the analyses presented in the present work is also provided as supporting information (see File S3).

**Table 2:** Number of genes considered per database.

Database name	alpha	cdc15	cdc28	alpha30	alpha38
**Genes from Alberghina et al. that also appear in the microarray database**	276	280	280	238	253
**Genes with missing data for one time point**	48	32	209	0	0
**Genes with missing data for two time points**	9	5	30	0	0
**Genes with missing data for more than two time points (excluded from the analysis)**	1	44	2	0	0
**Genes taken into account for the analysis**	275	236	278	238	253

The total number of genes reported by Alberghina et al. as cell cycle genes is 282.

## Results and Discussion

After the databases were transformed to make them comparable, they were individually analyzed using the SOM Toolbox [24], taking the genes as individuals (rows) and the expression levels at the time points as their properties (columns). An output map of 400 units (20×20) was created for each database in order to observe the way genes clustered according to their expression levels along all the time sequence. It should be noted that the total number of units determines the degree of separation or clustering of genes on the map. The fewer the number of units, the tighter the clusters are on the map, but with the risk of grouping together genes that could have no real relationship among them. Conversely, the higher the number of units, the more dispersed the clusters are on the map, with the risk of separating genes that could be related. For the present work, the quantity of units or clusters was determined empirically conducting several tests based on the *alpha* database, which contained a greater quantity of genes and had a smaller amount of missing data than the other databases (see Table 2). The determination of the number of units was based on the maximum similarity in the expression patterns of clustered genes. This number of units was applied to the other databases because we were interested in comparing the distances between neurons in which similar genes were located within the different databases. In order to improve the display quality of the output map, a hexagonal units grid was used, as suggested by Kohonen [25]. Clusters were visually inspected on the maps to detect patterns; we looked for clusters where the gene expressions included in all five databases were similar.

**Figure 2:**
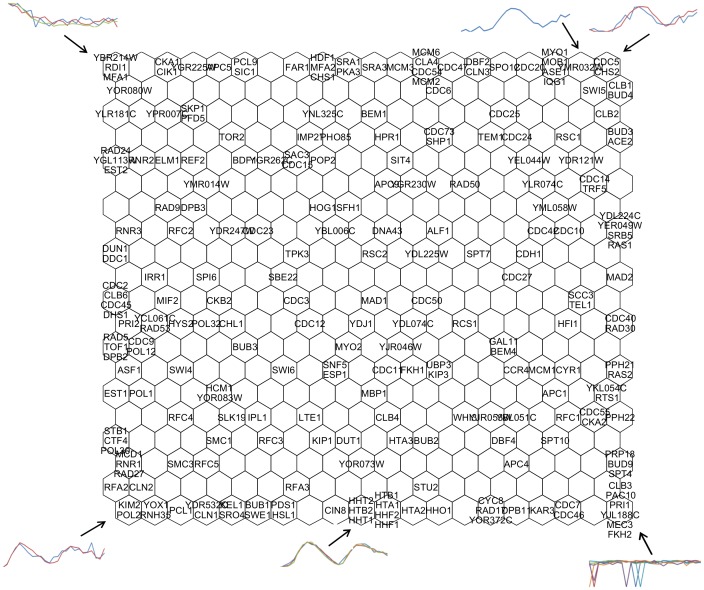
Output map containing the distributions of genes from the *alpha30* database. Genes are mapped according to their similarity in expression level. Gene clusters with similar expression levels in the time series are mapped to areas that are closer to each other. Arrows point to the neurons that correspond to the expression level graphs shown. Clusters that are closer on the map have more similar expression levels. Genes with different behavior are located farther away on the map. Furthermore, genes with opposite behavior tend to be located in opposite neurons on the map.

As an illustration, Figure 2 presents the output map for genes from the *alpha30* database. Genes with very similar expression levels along all time point tended to be assigned to the same neuron; moreover, the closer neurons were, the more similar the expression levels were in the gene clusters assigned to them. Conversely, gene clusters with different behavior were located farther away on the map; furthermore, genes with opposite behavior tended to be placed on opposite sides of the map. In Figure 2 many units are empty because the 238 genes selected to be analyzed from the *alpha30* database had to be distributed within some of the 400 units on the map, and thus it was inevitable that some of the units remained empty. If fewer units were used, the expression patterns of possibly unrelated genes could be grouped within the same cluster, leading to an incorrect classification of genes.

**Figure 3:**
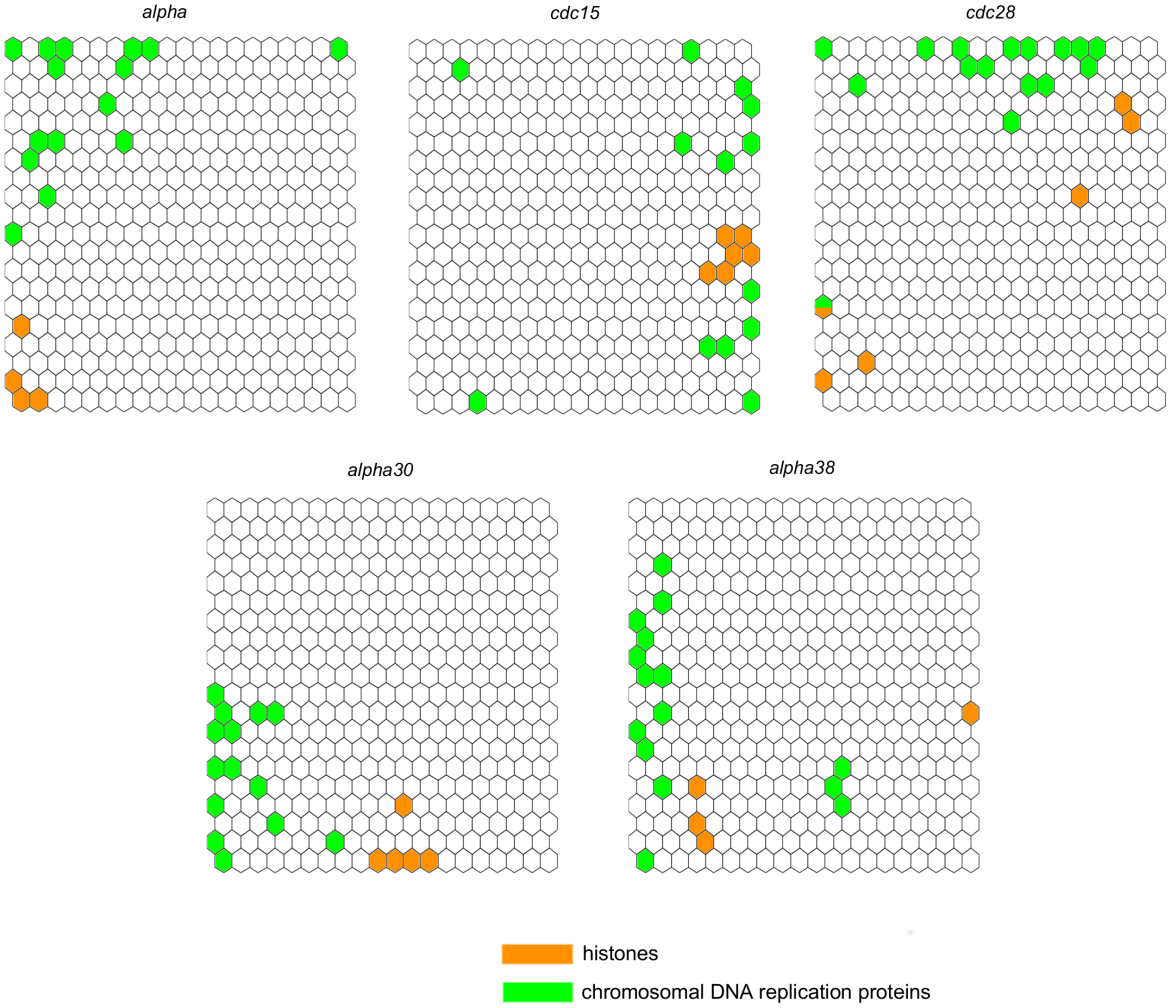
Location of genes with similar known biological functions on the output maps. Most histone encoding genes were clustered in neurons whose location was very close to each other, except in the case of the *cdc28* database. Most genes involved in chromosomal DNA replication were located in roughly the same neighborhood of each map, except in the case of the *cdc15* database.

In order to determine whether genes with known similar biological function were located in closer areas on the maps from all five databases, we looked for the histone and chromosomal DNA replication encoding genes mentioned in [18]. The histone genes were *hht1*, *hht2*, *hhf1*, *hhf2*, *hta1*, *hta2*, *htb1*, *hho1,* and *htb2*, whereas the genes involved in chromosomal DNA replication were *cdc9, ctf4, dbp2, est1, pri2, rfa2, rfa3, rfc4, rfc5, tof1, pol1*, *pol2*, *cdc2*, *pol12*, *pol30*, *pol31*, *hys2*, and *pol32*. Genes *ctf18, rfa1, tel2, top3 and ynk1* were not included in the present comparison because they are not included in the cell cycle genes described in [22]. As shown in Figure 3, on the maps from all the databases –with the exception of *cdc28–*, most histones were clustered in neurons that were very close together, indicating that in four of the five databases the expression patterns of histones were similar, whereas in the *cdc28* database, histones appeared to have different expression patterns along the two cell cycles. This same dissimilarity among the expression patterns in the *cdc28* database with respect to the *cdc15* and *alpha* databases is found in [18]. Most genes involved in chromosomal DNA replication were located in the same general region of each map –except in the case of *cdc15*. An explanation of these differences among expression patterns would most likely require wet-lab experiments to be carried out by biologists.

**Figure 4:**
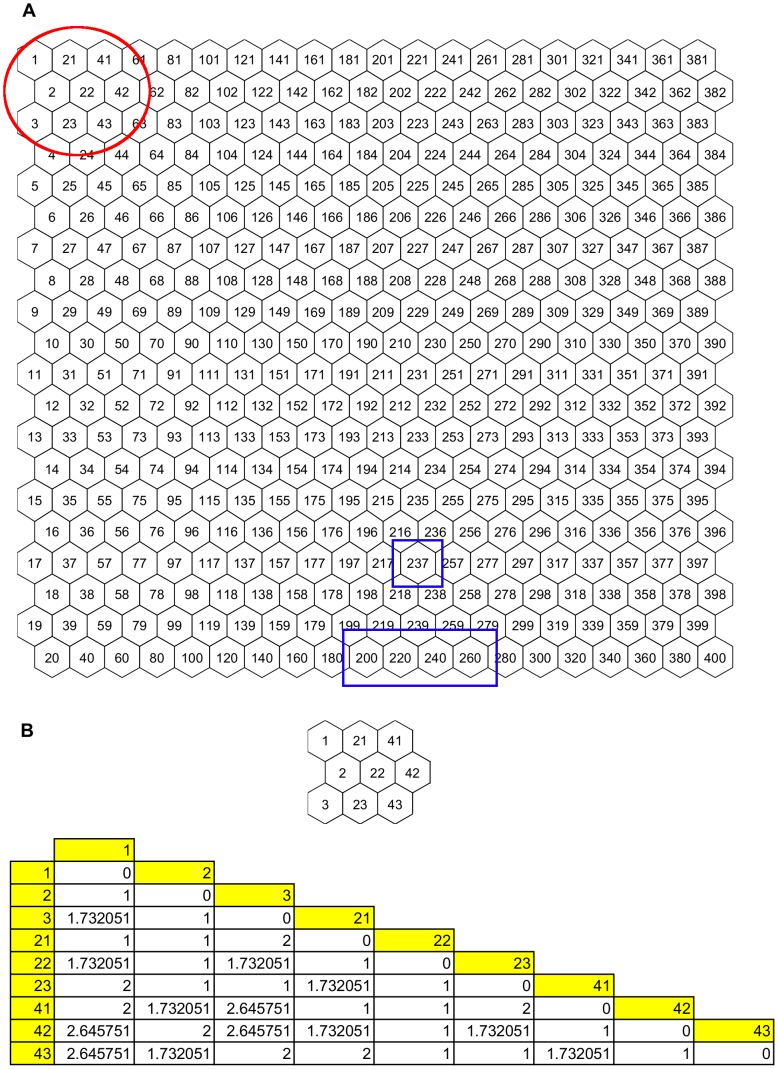
Neuron positions and distances between units on the output map. A: The 400 units on the output maps are labeled as shown. Histone encoding genes from the *alpha30* database are located in neurons 200, 220, 237, 240, and 260 (blue rectangles), meaning that they have a similar expression level behavior along the two cell cycles. B: Distance between the centroids of the units shown within the red circle in A.

Neuron positions on all output maps produced by SOM were numbered as shown in Figure 4A. This figure also shows in blue rectangles the neuron positions where the histone encoding genes from the *alpha30* database were placed on the map (neurons 200, 220, 237, 240, and 260). The assignment of genes to these adjacent neurons on the map means that they have a very similar expression level behavior along the two cell cycles. An illustration of the measured distances among the neurons shown within the red circle in Figure 4A is presented in Figure 4B. It should be noted that unit labels have no relation to the distance between units; e.g. the distance from unit 2 to unit 22 is only 1.

**Figure 5:**
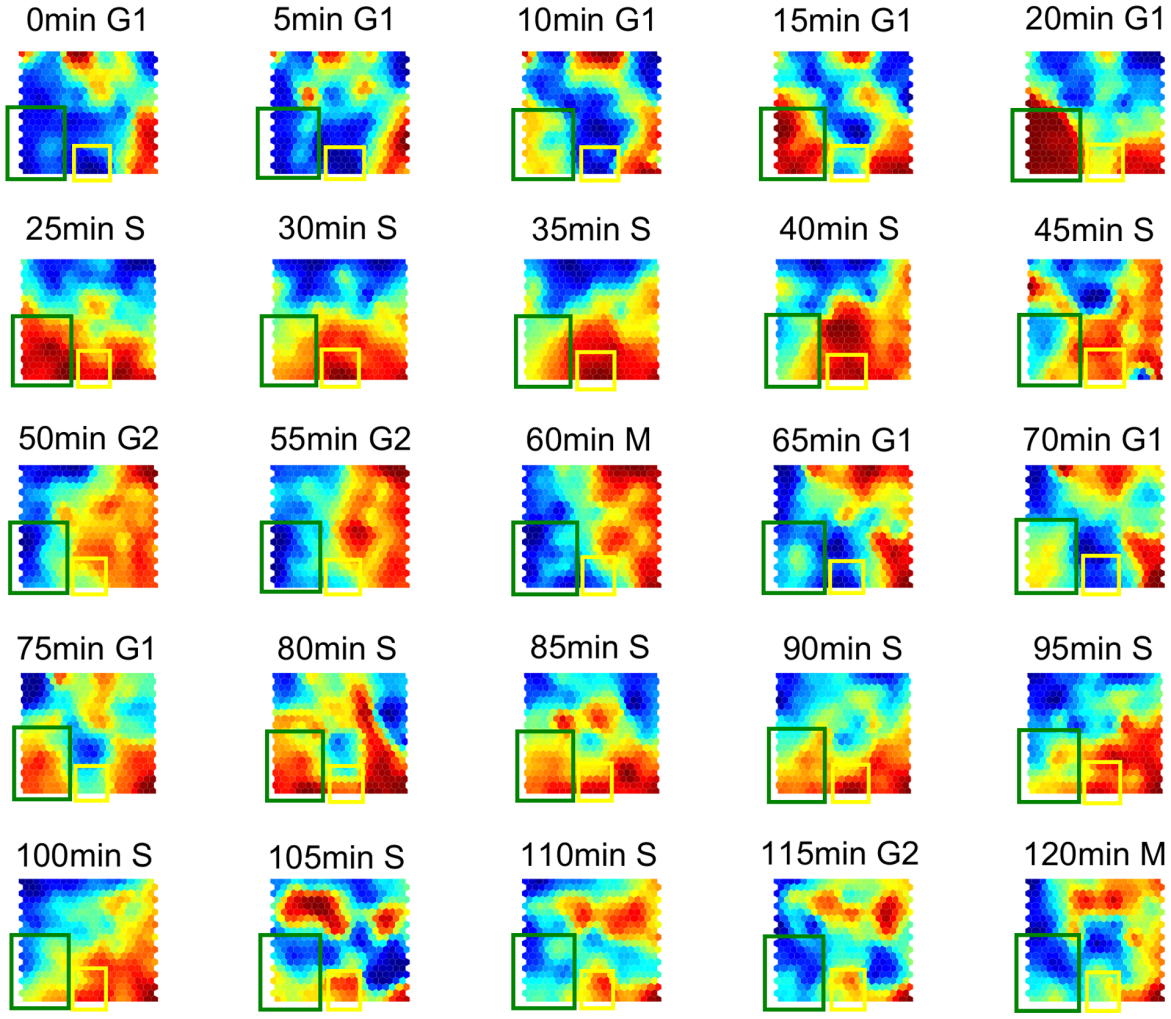
Color-coded output maps representing the final weight of neurons from the samples of the *alpha30* database. Maps are labeled with the sampling time and the cell cycle phase. The cluster centroid value is coded with a range of colors from blue for the lowest expression level value, to red for the highest value. Green rectangles enclose the region where the chromosomal DNA replication genes are located on the maps; these genes have their highest expression level during late G1 phase and early S phase. Yellow rectangles correspond to neurons with the genes coding for histones; these genes have their highest expression level during the S phase.

Output maps from SOM can also be colored to allow for a visual analysis of patterns. Figure 5 shows a series of maps with the final color-coded weight of neurons corresponding to the 25 samples from the *alpha30* database. The cluster centroid value is represented with colors from blue for the lowest expression level value to red for the highest value. Output maps are labeled in the figure with the time when the sample was taken and the cell cycle phase as reported by Pramila et al. [19]. In order to visually follow the evolution of expression levels of specific genes in time, it suffices to track the neurons where they are located along the series of colored maps. For instance, the green rectangle on the maps from Figure 5 encloses the neurons where chromosomal DNA replication genes are located, whereas the yellow rectangle surrounds the neurons corresponding to histone encoding genes. In this series of maps, it can be seen that histones, which are essential basic proteins that package the genomic DNA of all eukaryotes into nucleosomes to form chromatin [29], have their maximum expression during the S phase, in which DNA replication takes place. On the other hand, the maps from Figure 5 show that genes involved in chromosomal DNA replication have their maximum expression during late G1 phase and early S phase. As can be seen, color-coded output maps from SOM can be useful in the discovery of the biological function of genes by simply looking at the position of the cluster in which the genes are located and analyzing its behavior along the time series.

Through the use of SOM, it was also possible to detect the irregular behavior of genes under certain conditions. For instance, it was detected that in the *alpha30* database some genes that are reported as responsible for cell cycle regulation, such as *fkh2* and *clb3* located in unit 400 on the maps in Figure 5, do not present a cyclic behavior.

**Figure 6:**
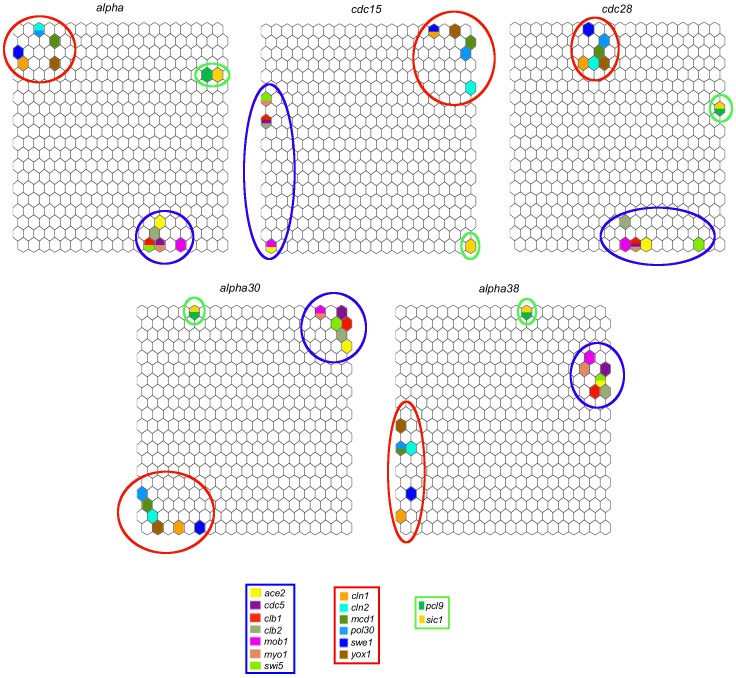
Genes clusters consistently located close to each other on the maps from the five databases. Some of these genes regulate each other either directly or indirectly according to the regulatory network reported by Alberghina et

Other genes whose corresponding neurons are consistently located close to each other on the maps were found, such as *ace2*, *clb1*, *clb2*, *mob1*, and *swi5*, as well as another group formed by *cln1*, *cln2*, *swe1*, and *yox1* (see Figure 6). According to the gene regulatory network published by Alberghina et al. [22], some of these genes regulate each other directly; for instance, *clb1* regulates *clb2*, *ace2* regulates both *swi5* and *clb2*, and *swe1* regulates *yox1*. Other genes have a reported indirect regulatory relationship; for example *ace2* regulates *mob1* through *clb3*, and *cln1* and *swe1* are regulated by *cdc28* [22]. We also found other pairs of genes that are consistently clustered very closely on the maps and which do not have an evident regulatory relationship in the reported network, such as *mcd1* and *pol30*, *cdc5* and *myo1*, and *pcl9* and *sic1* –except in the case of the *cdc15* database, which has no data for *pcl9*. The consistent proximity of the neurons corresponding to these genes on the maps suggests that they may have a more direct regulatory relationship not yet discovered by biological methods.

The analysis of genes for which the relationship among them is already known, provided us with elements to validate the usefulness of SOM to discover relationships, and its possible application to other databases containing genes for which the relationships among them is not known yet. However, the results obtained by this method are not conclusive. Wet-lab experiments would most likely be required to explain the clustering tendencies described in the present paper. It should also be noted that the objective of this work is not finding relationships solely among genes in the yeast, but to show the usefulness of SOM in the analysis of biological databases, such as those derived from expression microarrays, in order to find relationships among genes.

**Figure 7:**
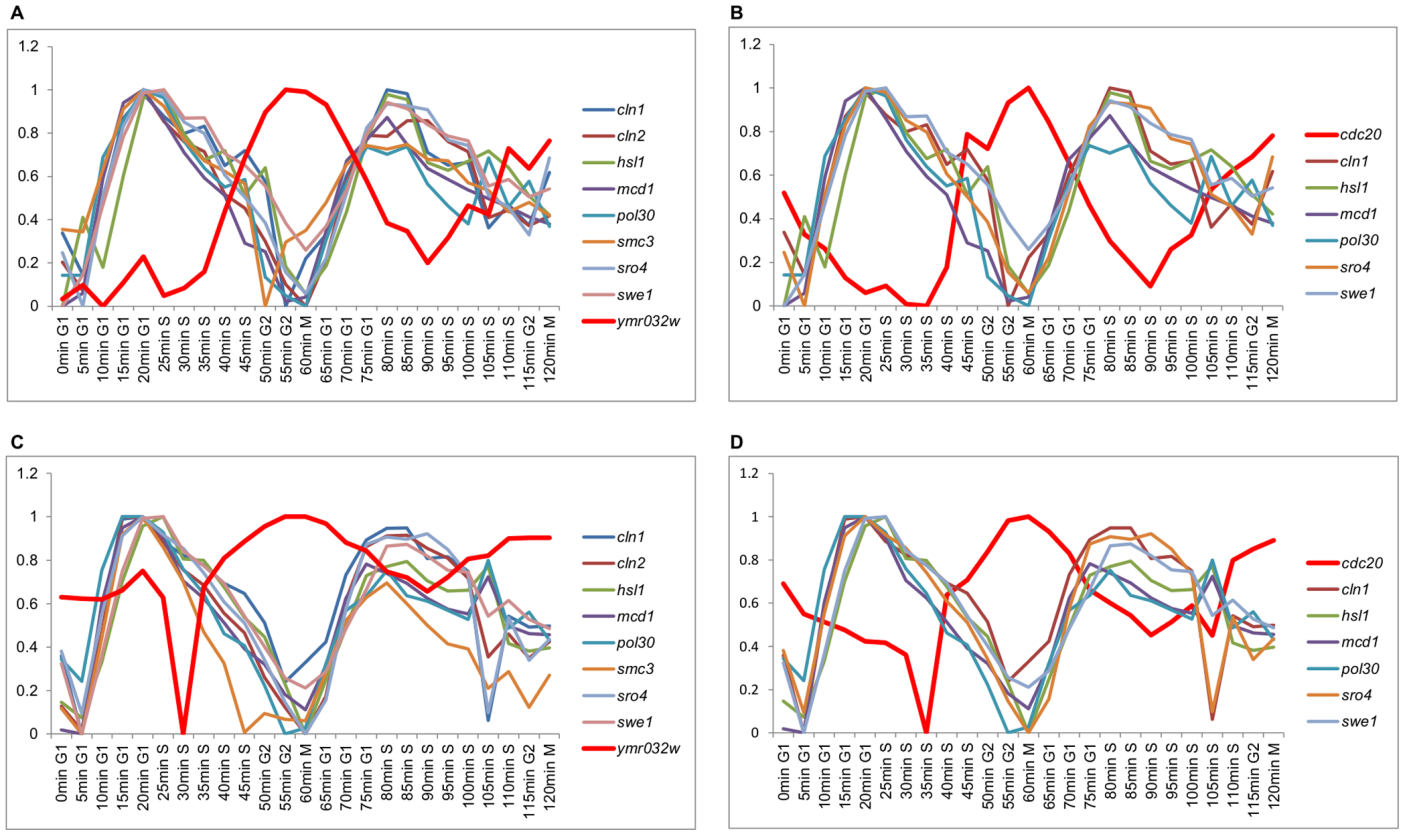
Examples of genes that are consistently located far from each other. Expression level graphs for all samples for genes from the *alpha30* and *alpha38* databases. The abscissa coordinate labels contain the sampling time and the cell cycle phase. A: The *ymr032w* gene has an approximate opposite behavior to the other genes shown from the *alpha30* database, which have a similar expression level behavior among them. B: The *cdc20* gene shows a roughly opposite behavior to the other genes shown from the *alpha30* database. C: Same as in A, but with genes from the *alpha38* database. D: Same as in B, but with genes from the *alpha38* database.

Genes consistently located far from each other were also identified, suggesting a behavior that could correspond to a negative regulatory relationship. Figure 7 shows instances of this behavior in genes from the *alpha30* and *alpha38* databases. Gene *ymr032w* in Figures 7A (*alpha30*) and 7C (*alpha38*) shows roughly an opposite behavior to the other genes shown; similarly, gene *cdc20* in Figures 7B (*alpha30*) and 7D (*alpha38*) shows an approximate opposite behavior to the other genes shown. As an illustration, a relationship between *cdc20* and *hsl1* is reported in the regulatory network from Alberghina et al. [22]. Furthermore, it has been reported that *cdc20* negatively regulates *hsl1* [30], as suggested by the opposite behavior in their expression levels found by the SOM. These results might be used as a starting point for the study of the association among the genes mentioned for which a direct relationship has not yet been reported.

In order to verify whether there were genes that could have been affected during the experimental conditions under which the microarray databases were obtained, the distances between pairs of the genes clustered in all databases were calculated with the goal of finding outliers. We used the distance between the hexagons from the output map to make the outliers computations, which were based on the quartiles of the data as calculated using Eq. 4.
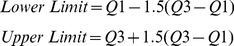
(4)


**Figure 8:**
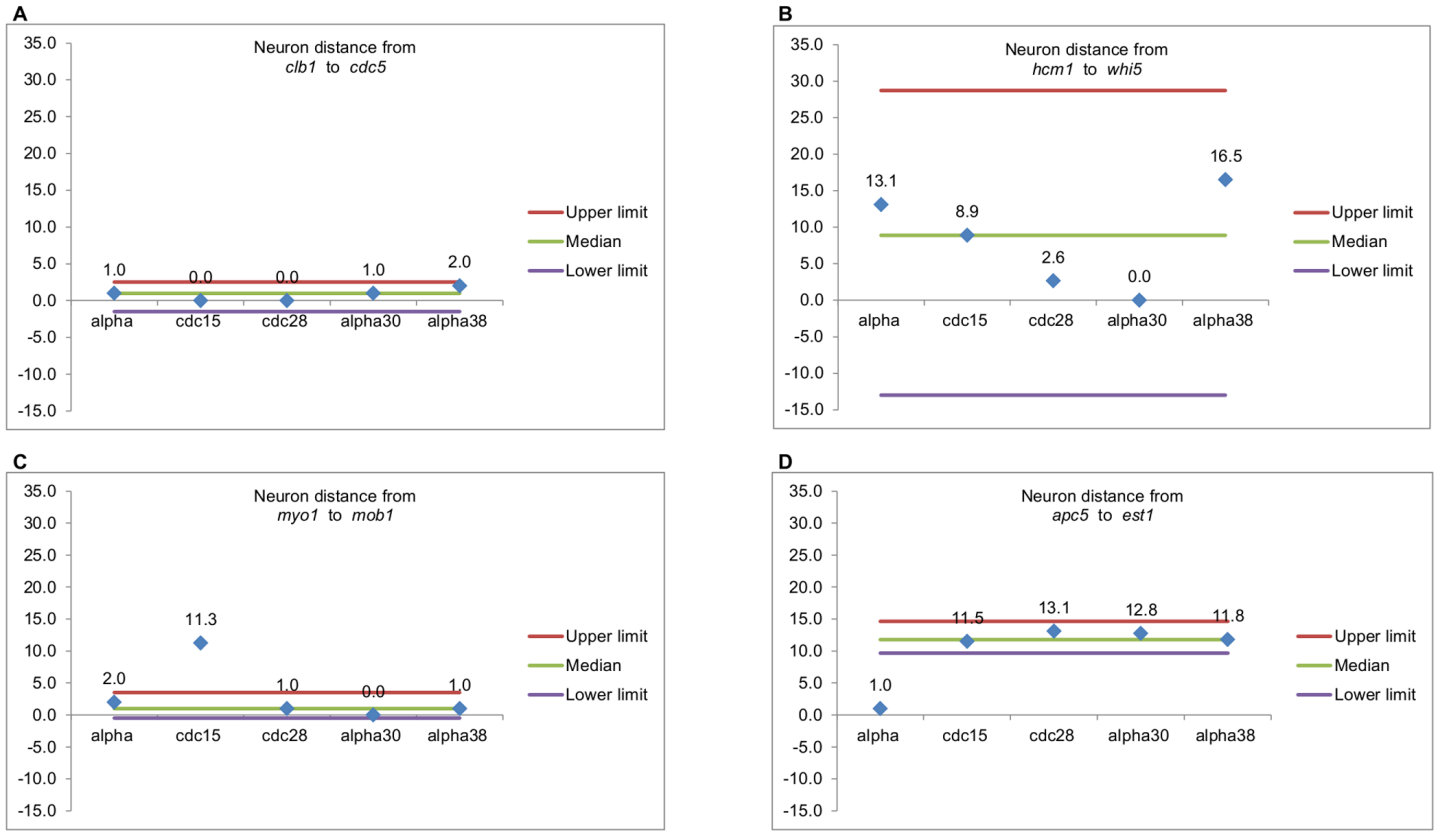
Examples of analyses of distance between pairs of genes. Genes in A are consistently close in all the databases, meaning that their behavior is similar in all of them, whereas distances between genes in B are very different in all the databases. The pairs of genes in C and D have different behavior in one of the five databases, suggesting that they may have been affected by the experimental conditions for obtaining that particular database.

The database that presented the fewest outliers was *alpha30*, whereas the database that presented the highest number of outliers was *cdc28* (see Table 3). Figure 8 presents some examples of the analyzed cases. Figure 8A shows that genes *clb1* and *cdc5* were consistently close in all the databases, meaning that their behavior was similar in all of them, whereas distances between genes in Figure 8B are very different in all the databases, indicating that *hcm1* and *whi5* presented different expression levels during the two cell cycles analyzed. Figures 8C and 8D show pairs of genes that have different behavior in one of the five databases, which suggest that they may have been affected by the experimental conditions for obtaining that particular database.

**Table 3:** Number of outlier gene pairs per database.

Database name	alpha	cdc15	cdc28	alpha30	alpha38
**Number of outlier gene pairs**	326	319	375	217	289

Only genes with data for all five databases were analyzed. The total number of gene pairs analyzed was 17755 for all databases.

The above analysis shows that the behavior of genes in experiments performed under different conditions or with different strains of *S. cerevisiae* is not similar in all cases. This could be one reason why after more than fifteen years and hundreds of research papers on the genetic regulatory network of *S. cerevisiae*, it has been difficult to find a consensus among researchers about the way in which genes interact in this organism. Additionally, taking into account that the time of transcription and translation of genes is in the order of minutes or even seconds [31], a more frequent sampling might be needed to capture the dynamics of gene regulation [12].

Self-Organizing Maps can be useful in the analysis of gene expression levels from microarray data, since in addition to clustering the genes according to their behavior over time, they organize and display the clustered genes based on their behavior. For example, Self-Organizing maps place the genes with the highest similarity in nearby neurons, and the ones with lower similarity in neurons that are farther away on the map. Another advantage of this kind of neural network is that it is an unsupervised method; thus, there is no need of previous knowledge to perform the analysis. This property of not being supervised, allows SOMs to avoid certain amount of bias by the researcher when extracting the possible model from the data. This is one of the main reasons why SOM, as other unsupervised techniques, may be a better choice than other techniques for obtaining more accurate models of biological processes.

It should finally be mentioned that the computational complexity of the SOM algorithm is *O*(*K* ^2^), where *K* is the number of map units. However, it is possible to reduce the complexity by using parallel versions of SOM [32], [33]. The running time for the analysis of each of the studied databases was around three seconds in a computer with an Intel Core i5 2.5-GHz processor, 4 GB of RAM, and a 64-bit Windows 7 operating system.

## Conclusions

In this paper we propose the use of Self-Organizing Maps for analyzing the similarity of gene expression levels from DNA microarray databases. We applied the SOM technique to five of the most studied microarray databases of *Saccharomyces cerevisiae*. Through this tool it was possible to discover possible relationships among genes, based on their behavior over time in all the databases, without any previous knowledge about their characteristics, such as biological functions or their binding sites. Furthermore, it was possible to find genes that behaved in similar ways in all databases and others that behaved in a different way in just one of the databases, suggesting that they could be affected by the experimental conditions or the yeast strain used. We also showed the usefulness of visualization of expression levels through color-coded maps in order to analyze the evolution of gene expression levels during the cell cycle. This type of maps could be useful in the discovery of new biological functions of genes according to the cell cycle phase in which their expression level peaks.

The proposed method can also assess the quality of the expression microarray database analyzed. For instance, we found that of the five databases that were analyzed, the one with the best characteristics to study the gene regulatory network of *S. cerevisiae* seemed to be the *alpha30* database. This database presented the fewest pairs of outlier genes in our comparative analysis with SOM, which suggests that this database might be the most representative of the dynamics of gene regulation of the five databases considered. In this database, as in the *alpha38* database, samples were taken every five minutes, –a sampling time shorter than in the other databases– and they contained 25 samples –more samples than the others.

The methodology presented in this paper could be applied to the study of the relationships among genes in other organisms and biological processes other than cell cycle regulation, using the expression levels in time series microarray data as input. It could be particularly useful in situations where there is little experimental knowledge about the biological process of interest. The output maps from SOM could be the starting point for laboratory-based experiments aimed at discovering how genes interact in complex regulatory networks.

## Supporting Information

Dataset S1 **The *alpha* database.** SOM Toolbox data file corresponding to the *alpha* database with expression values scaled to the range [0,1].
(DATA)

Dataset S2 **The *cdc15* database.** SOM Toolbox data file corresponding to the *cdc15* database with expression values scaled to the range [0,1].
(DATA)

Dataset S3 **The *cdc28* database.** SOM Toolbox data file corresponding to the *cdc28* database with expression values scaled to the range [0,1].
(DATA)

Dataset S4 **The *alpha30* database.** SOM Toolbox data file corresponding to the *alpha30* database with expression values scaled to the range [0,1].
(DATA)

Dataset S5 **The *alpha38* database.** SOM Toolbox data file corresponding to the *alpha38* database with expression values scaled to the range [0,1].
(DATA)

File S1 **List of the cell cycles genes from Alberghina et al. in XLS format.**
(XLS)

File S2 **List of the cell cycles genes from Alberghina et al. in CSV format.**
(CSV)

File S3 **Compressed file with the SOM Toolbox files used for the analyses.**
(ZIP)
